# Immunolocalization of Kisspeptin Associated with Amyloid-*β* Deposits in the Pons of an Alzheimer's Disease Patient

**DOI:** 10.1155/2013/879710

**Published:** 2013-05-16

**Authors:** Amrutha Chilumuri, Maria Ashioti, Amanda N. Nercessian, Nathaniel G. N. Milton

**Affiliations:** ^1^Department of Human and Health Sciences, School of Life Sciences, University of Westminster, 115 New Cavendish Street, London W1W 6UW, UK; ^2^Health Sciences Research Centre, University of Roehampton, Holybourne Avenue, London SW15 4JD, UK

## Abstract

The pons region of the Alzheimer's disease (AD) brain is one of the last to show amyloid-*β* (A*β*) deposits and has been suggested to contain neuroprotective compounds. Kisspeptin (KP) is a hormone that activates the hypothalamic-pituitary-gonadal axis and has been suggested to be neuroprotective against A*β* toxicity. The localization of KP, plus the established endogenous neuroprotective compounds corticotropin releasing hormone (CRH) and catalase, in tissue sections from the pons region of a male AD subject has been determined in relation to A*β* deposits. Results showed A*β* deposits also stained with KP, CRH, and catalase antibodies. At high magnification the staining of deposits was either KP or catalase positive, and there was only a limited area of the deposits with KP-catalase colocalization. The CRH does not bind A*β*, whilst both KP and catalase can bind A*β*, suggesting that colocalization in A*β* deposits is not restricted to compounds that directly bind A*β*. The neuroprotective actions of KP, CRH, and catalase were confirmed *in vitro*, and fibrillar A*β* preparations were shown to stimulate the release of KP *in vitro.* In conclusion, neuroprotective KP, CRH, and catalase all colocalize with A*β* plaque-like deposits in the pons region from a male AD subject.

## 1. Introduction

The deposition of the amyloid-*β* (A*β*) peptide within plaques in the Alzheimer's disease (AD) brain is a central feature of the disease pathology [1, 2]. A sequential pattern of A*β* deposition within different regions of the brain has been suggested as AD progresses [3–6]. The staging of A*β* deposition by Thal et al. (2002) [3] identified the cerebellum plus brainstem nuclei including the pons as the last to show A*β* deposits. In transgenic mice overexpressing the human amyloid precursor protein (APP) the A*β* deposition showed a similar sequential pattern, with the cerebellum and pons again the last to show A*β* deposits [7]. The apparent resistance of the cerebellum and pons to neurodegenerative changes suggests that endogenous neuroprotective processes may play a role in these tissues.

A range of endogenous compounds have been suggested to have neuroprotective properties against A*β* in AD models [8–16]. In a recent study kisspeptin (KP) peptides were suggested to have neuroprotective properties against A*β* plus related amyloid proteins [17]. The KP peptide is a reproductive hormone [18], and the female hypothalamic levels of KP show elevations after menopause that are not seen in males [19]. Female AD onset is typically postmenopausal, and there is significantly less neurodegeneration in the hypothalamus in women compared to men [20]. The release of KP from human neuronal cells has been shown to be stimulated by A*β* [17] suggesting that in regions that express the KiSS-1 gene, which encodes for the KP peptides, there may be changes in KP levels in AD due to the elevations of A*β*. 

The KP peptide was identified as having similarity to the catalase region that binds A*β* [21], and KP binds A*β* itself [17]. Catalase has been shown to bind directly to A*β* fibrils [22] and has been found by immunohistochemistry in amyloid plaques in AD brains [23]. The catalase enzyme has been shown to have neuroprotective properties as an antioxidant enzyme [24, 25], as an A*β* binding protein [26, 27] and when targeted to the mitochondria as a modifier of A*β* secretion [12]. The CRH peptide has well-established neuroprotective properties and prevents A*β* toxicity [10, 28–33]. The mechanism for CRH neuroprotection, unlike KP neuroprotection, is receptor mediated [30–32], and the peptide does not bind A*β*, unlike catalase and KP [17, 26]. In AD the CRH peptide has been found to be associated with thioflavin S-positive deposits [34], and the levels of CRH are markedly reduced in some brain regions [35, 36].

The KP peptide [37, 38], CRH peptide [39], and catalase [40, 41] are all found in the pons, where neurodegenerative changes and A*β* deposition occur in the latter stages of the disease [3, 7]. In this study the localization of immunoreactive (ir-) KP, CRH, and catalase in relation to A*β* deposits has been determined in pons sections from a male AD patient. The neuroprotective effects of KP, CRH, and catalase plus ir-KP release from human SH-SY5Y neuroblastoma cells have also been studied. 

## 2. Materials and Methods

### 2.1. Materials

Pons sections from a 72-year-old male with AD (Cat. no. ab4586; Lot no. B506287) and a 26-year-old normal male (Cat. no. ab4316; Lot no. A504234) and BAM-10 mouse anti-A*β* antibody were obtained from Abcam PLC, Cambridge, UK. Rabbit anti-KP 45–54 antiserum, KP 1–54, KP 42–54, KP 45–54, KP 45–50, neuropeptide-FF (NPFF), CRH, A*β* 1–46, A*β* 1–43, A*β* 1–42, A*β* 1–40, A*β* 1–38, and A*β* 17–40 were purchased from Bachem AG, Switzerland. Goat anti-mouse IgG Alexa Fluor 568 and goat anti-rabbit IgG Alexa Fluor 488 were purchased from Chemicon, UK. VECTASHIELD Mounting Media was purchased from Vector Laboratories Ltd., UK. The CAT-505 mouse anti-catalase antibody, alkaline phosphatase conjugated goat anti-rabbit IgG, alkaline phosphatase conjugated anti-mouse IgG, and all other chemicals were purchased from Sigma-Aldrich, UK.

### 2.2. A*β* Fibril Formation

Batches of synthetic A*β* 1–46, A*β* 1–43, A*β* 1–42, A*β* 1–40, A*β* 1–38, A*β* 17–40, or A*β* 25–35 were dissolved in distilled water at a concentration of 1.0 mg/mL and incubated at 37°C for 24 h, with constant oscillation. Following incubation, the formation of fibrils was confirmed by TEM or Congo red assay as previously described by Milton and Harris [22, 42, 43].

### 2.3. Antibody Characterization

NUNC MaxiSorp 96-well immunoplates were coated with 1 *μ*g/mL of either KP peptides, NPFF peptides, CRH peptides, catalase, or A*β* peptides in 50 mM carbonate buffer, pH 9.6, and unoccupied sites blocked with 0.2% (w/v) marvel. Either the BAM-10 mouse anti-A*β* antibody [44], rabbit anti-A*β* 21–32 antiserum [45], rabbit anti-KP 45–54 antiserum [17], KCHMB001 mouse anti-CRH antibody [46–48], or CAT-505 mouse anti-catalase antibody [49] at a final concentration of 0.1 *μ*g/mL in 50 mM TRIS (containing 0.1% BSA and 0.1% Triton X-100) was added (100 *μ*L/well) and incubated at 4°C for 16 hours. Some plates were coated with A*β* 1–42 fibrils, prepared, and tested as described by Milton and Harris (2009) [22], and these plates were preincubated at 4°C for 24 hours with KP 45–54, NPFF, CRH or catalase prior to addition of antibodies. After washing to remove unbound material an alkaline phosphatase conjugated goat anti-rabbit or anti-mouse secondary antibody was added and incubated at 24°C for 2 hours. After washing to remove unbound material p-nitrophenylphosphate substrate was added and absorbance at 405 nm determined. 

### 2.4. Immunohistochemistry and Confocal Microscopy

Pre-mounted and paraffin-embedded pons tissue sections from a normal or an AD patient at a thickness of 5 *μ*M were used. The tissue had been examined and diagnosed by a licensed pathologist and was ethically obtained. The sections were processed for immunostaining [50] and incubated overnight at 4°C with 1 : 1000 dilutions (1 *μ*g/mL final concentration) of BAM-10 mouse anti-A*β* antibody [44], rabbit anti-A*β* 21–32 antiserum [45], rabbit anti-KP 45–54 antiserum [17], KCHMB001 mouse anti-CRH antibody [46–48], and CAT-505 mouse anti-catalase antibody [49] in phosphate buffered saline containing 0.01% Tween 20 (PBST). For some of the incubations with anti-KP 45–54 the antibody solutions were preincubated with NPFF (10 *μ*g/mL) for 24 h to block binding to endogenous NPFF [51]. The sections were then washed for 3 × 5 min with PBST before the secondary antibodies were applied (goat anti-mouse IgG-Alexa Fluor 568 and goat anti-rabbit IgG-Alexa Fluor 488, 1 : 500) for 1 hour. The sections were then washed in PBST, and cover slips were mounted with VECTASHIELD Mounting Media.

Images were acquired by sequential scanning using a Leica TCS SP2 confocal system (Leica Microsystems, Milton Keynes, UK) with a 63x ceramic dipping objective. A 488 nm laser was used for excitation of Alexa Fluor 488 labeled goat anti-rabbit IgG, while a 543 nm laser was used for Alexa Fluor 568 labeled goat anti-mouse IgG excitation [52].

### 2.5. Cell Cultures

Human SH-SY5Y neuroblastoma cells were routinely grown in a 5% CO_2_ humidified incubator at 37°C in a 1 : 1 mixture of Dulbecco's modified Eagle's medium and HAM's F12 with Glutamax (Invitrogen) supplemented with 10% fetal calf serum (FCS), 1% nonessential amino acids, penicillin (100 units/mL), and streptomycin (100 mg/mL) [53]. Human neuroblastoma SH-SY5Y cells were cultured in 6-well or 96-well plates and differentiated with retinoic acid for 7 days prior to experimentation.

### 2.6. KP Release

2.5 × 10^5^ differentiated SH-SY5Y cells/well in 6-well plates were incubated in 4 mL of medium containing a subtoxic dose (100 nM) of either fibrillar A*β* 1–46, A*β* 1–43, A*β* 1–42, A*β* 1–40, A*β* 1–38, A*β* 17–40, or A*β* 25–35 for 4 h. Control cells were cultured in medium alone. Media was harvested and KP extracted using a polyclonal anti-KP 45–54 antiserum and a protein-A agarose column. The immunoreactive KP was eluted from the column in 0.5 M acetic acid and was further purified using a Sep-Pak C_18_ extraction step. The Sep-Pak C_18_ columns were prewetted with methanol and 0.5 M acetic acid, acidified samples applied, and columns washed with 0.5 M Acetic acid prior to elution of bound peptide with 70% acetonitrile. After drying under a stream of nitrogen, samples were resuspended in PBS containing 0.1% BSA plus 0.05% Tween 20. 

### 2.7. Determination of ir-KP

ELISA plates were coated with 1 *μ*g/mL anti-KP 45–54 antiserum in 50 mM carbonate buffer, pH 9.6, and unoccupied sites blocked with 5% (w/v) marvel. Samples or synthetic KP 45–54 standards (0–1000 pg/mL) were applied in assay buffer (PBS containing 0.1% BSA plus 0.05% Tween 20) and incubated for 24 h. After washing with assay buffer to remove unbound material, biotinyl-KP 45–50 (10 ng/mL) was added and incubated for 2 h. After washing to remove unbound material, immunoreactive- (ir-) KP-like material was detected using a streptavidin-horseradish peroxidase conjugate and 3,3′,5,5′-tetramethylbenzidine substrate [17]. Sample levels were compared to KP 45–54 standards and ir-KP-like peptide levels expressed as a % of control cell release.

### 2.8. Effect of KP, CRH, and Catalase on A*β* Neurotoxicity

On the day of the experiment 5 × 10^3^ differentiated SH-SY5Y cells/well in 96-well plates were pretreated with either media alone (control) or anti-KP 45–54 antibody (10 *μ*g/mL) to block endogenous KP, KCHMB001 anti-CRH antibody (10 *μ*g/mL) to block endogenous CRH, or 3-aminotriazole (3AT: 50 *μ*M) to inhibit endogenous catalase [24, 53] for a 4 h period. The A*β* 1–42 (10 *μ*M) was then added to induce toxicity, and cells were incubated for 16 hours prior to determination of cell viability. In experiments to assess the neuroprotection by KP, CRH, or catalase the KP 1–54 (10 *μ*M), CRH (10 nM), or catalase (5 *μ*g/mL) was incubated for 4 h with the A*β* 1–42 (10 *μ*M) to allow binding to occur prior to addition to cells. The mixtures of A*β* 1–42 plus either media alone or KP 1–54, CRH, or catalase were added to cells to induce toxicity and incubated for 16 hours prior to determination of cell viability.

### 2.9. Cell Viability

After treatment with test peptides or drugs and incubation for the appropriate time, the viability was determined by MTT reduction [21]. After incubation with test substances, MTT (10 *μ*L : 12 mM stock) was added and cells incubated for a further 4 hours. Cell lysis buffer (100 *μ*L/well; 20% (v/v) SDS, 50% (v/v) N,N-dimethylformamide, pH 4.7) was added, and after repeated pipetting to lyse cells, the MTT formazan product formation was determined by measurement of absorbance change at 570 nm. Control levels in the absence of test substances were taken as 100% and the absorbance in the presence of cells lysed with Triton X-100 at the start of the incubation period with test substances taken as 0% [54].

### 2.10. Data Analysis

All data are expressed as means ± SEM for ir-KP measurements levels in samples were determined from a standard curve using synthetic KP 45–54 as the standard. For cytotoxicity experiments data are expressed as % control cells (MTT reduction). Statistical analysis was performed by one-way analysis of variance (ANOVA) with Tukey or Dunnett multiple comparison post hoc testing using GraphPad Prism software (version 6), with a *P* value of <0.05 considered statistically significant.

## 3. Results

### 3.1. Characterization of Antibodies

**Figure 1:**
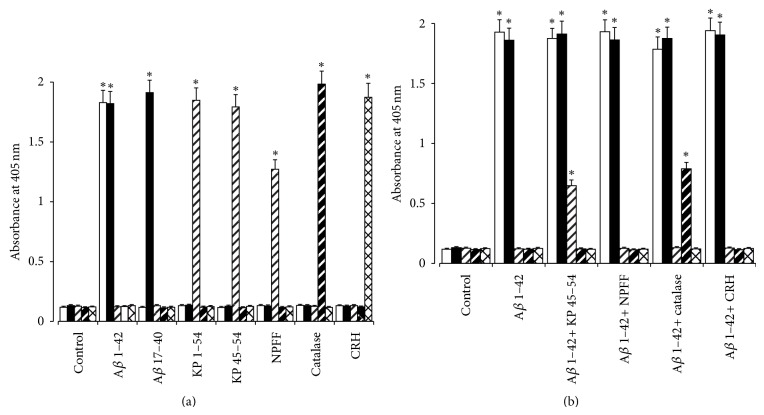
Binding of anti-A*β*, anti-KP, anti-catalase and anti-CRH antibodies to A*β*, KP, NPFF, catalase and CRH. In (a) plates were coated with A*β* 1–42, A*β* 17–40, KP 1–54, KP 45–54, NPFF, catalase, and CRH. Binding of BAM-10 mouse anti-A*β* antibody (open columns), rabbit anti-A*β* 21–32 antiserum (closed columns), rabbit anti-KP 45–54 antiserum (open hatched columns), CAT-505 mouse anti-catalase antibody (closed hatched columns) and KCHMB001 mouse anti-CRH antibody (open cross-hatched columns) was detected using alkaline phosphatase conjugated secondary antibodies and p-nitrophenylphosphate substrate. In (b) plates coated with A*β* 1–42 fibrils were preincubated with KP 45–54, NPFF, catalase, or CRH before addition of BAM-10 mouse anti-A*β* antibody (open columns), rabbit anti-A*β* 21–32 antiserum (closed columns), rabbit anti-KP 45–54 antiserum (open hatched columns) and CAT-505 mouse anti-catalase antibody (closed hatched columns), KCHMB001 mouse anti-CRH antibody (open cross-hatched columns). Binding was detected using alkaline phosphatase conjugated secondary antibodies and p-nitrophenylphosphate substrate. Results are expressed as mean ± SEM (*n* = 8) absorbance change at 405 nm. (∗ = *P* < 0.05 versus control; one-way ANOVA).

The binding of BAM-10 mouse anti-A*β* antibody [44], rabbit anti-A*β* 21–32 antiserum [45], rabbit anti-KP 45–54 antiserum [17], CAT-505 mouse anti-catalase antibody [49], and KCHMB001 mouse anti-CRH antibody [46] to A*β*, KP, NPFF, catalase, and CRH was tested. Both of anti-A*β* antibodies, BAM-10 mouse anti-A*β* and rabbit anti-A*β* 21–32, showed significant binding to full length A*β* but not to KP, NPFF, catalase, or CRH (Figure 1(a)). The BAM-10 antibody did not bind A*β* 17–40 in agreement with the published specificity of this antibody for A*β* 1–12 [44, 55, 56], whilst the anti-A*β* 21–32 antibody showed significant binding to A*β* 17–40. The anti-KP 45–54 antibody showed significant binding to KP 1–54 plus KP 45–54 and also showed significant binding to NPFF but did not cross-react with A*β*, catalase, or CRH (Figure 1(a)). The CAT-505 anti-catalase antibody showed significant binding to catalase and showed no cross-reactivity with A*β*, KP, NPFF, or CRH peptides. The KCHMB001 anti-CRH antibody showed significant binding to CRH and showed no cross-reactivity with A*β*, KP, NPFF or catalase.

The BAM-10 anti-A*β*, anti-A*β* 21–32, and anti-KP 45–54 antibodies all showed significant binding to plates coated with A*β* 1–42 fibrils and pretreated with KP 45–54 (Figure 1(b)). The BAM-10 anti-A*β* and anti-A*β* 21–32 antibodies but not the anti-KP 45–54 antibody showed significant binding to plates coated with A*β* 1–42 fibrils and pretreated with NPFF (Figure 1(b)). This observation contrasts with the cross-reactivity of the anti-KP 45–54 antibody with NPFF (Figure 1(a)) and suggests either that no specific binding of NPFF to A*β* had occurred or that the epitope of NPFF recognized by the antibody is inaccessible for antibody binding when the NPFF is bound to A*β*. The CAT-505 anti-catalase antibody showed no significant binding to plates coated with A*β* 1–42 fibrils and pretreated with either KP 45–54 or NPFF (Figure 1(b)). The BAM-10 anti-A*β*, anti-A*β* 21–32, and CAT-505 anti-catalase antibodies showed significant binding to plates coated with A*β* 1–42 fibrils and pretreated with catalase (Figure 1(b)). The anti-KP 45–54 antibody showed no binding to plates coated with A*β* 1–42 fibrils and pretreated with catalase (Figure 1(b)). The KCHMB001 anti-CRH antibody showed no significant binding to plates coated with A*β* 1–42 fibrils or those pretreated with either CRH, KP 45–54, NPFF, or catalase (Figure 1(b)), suggesting that either no specific CRH binding to A*β* had occurred or that the epitope of CRH recognized by the antibody is inaccessible for antibody binding when the CRH is bound to A*β*. The BAM-10 anti-A*β* and anti-A*β* 21–32 antibodies showed significant binding to plates coated with A*β* 1–42 fibrils and pretreated with CRH (Figure 1(b)).

### 3.2. Double-Labeling Immunohistochemistry for KP and A*β* in a Normal Control

**Figure 2:**
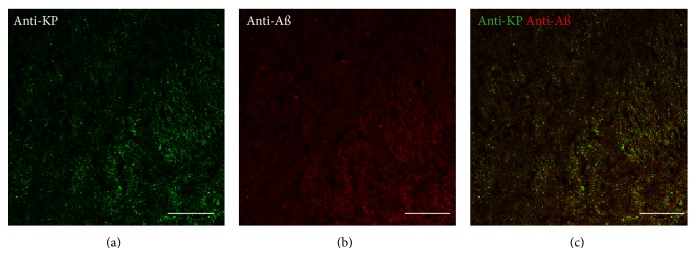
Double-labeling immunofluorescence demonstrating colocalization of kisspeptin (KP) and amyloid-*β* (A*β*) in the pons of a normal control (a–c). KP appears green, and A*β* appears red. The overlap of KP and A*β* appears yellow. Bars = 50 *μ*m.

Immunohistochemistry analysis with anti-KP 45–54 alone in pons sections from a 26-year-old normal male (Cat. no. ab4316; Lot no. A504234) showed staining that was detectable with green fluorescence but not red fluorescence. Immunohistochemistry analysis with BAM-10 anti-A*β* alone showed staining that was detectable with red fluorescence but not green fluorescence. Control incubations with secondary antibodies showed no detectable staining above background. The results from the double-labeling immunohistochemistry showed staining of tissue with the anti-KP 45–54 (Figure 2(a)) and BAM-10 anti-A*β* (Figure 2(b)) antibodies. There was no colocalization of anti-KP 45–54 and BAM-10 anti-A*β* labeling observed in the tissue (Figure 2(c)). 

### 3.3. Double-Labeling Immunohistochemistry for KP and A*β* in AD

**Figure 3:**
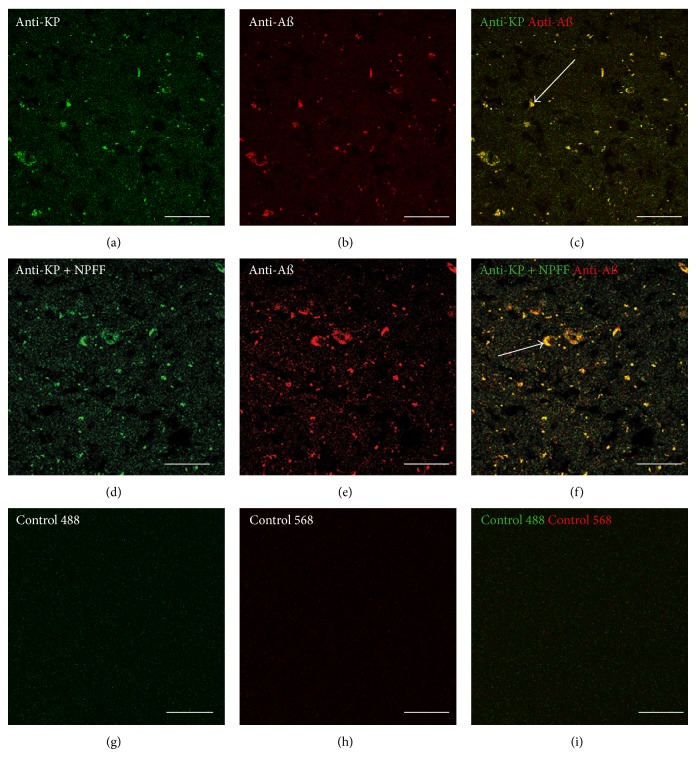
Double-labeling immunofluorescence demonstrating colocalization of kisspeptin (KP) and amyloid-*β* (A*β*) in the pons in Alzheimer's disease (a–f). Staining due to nonspecific binding of secondary antibodies is shown in (g–i). KP appears green, and A*β* appears red. The overlap of KP and A*β* appears yellow, and examples are labeled with arrows in (c) and (f). Bars = 50 *μ*m.

The results from the double-labeling immunohistochemistry in the pons sections from a 72-year-old male with AD (Cat. no. ab4586; Lot no. B506287) show that the anti-KP 45–54 (Figure 3(a)) and BAM-10 anti-A*β* (Figure 3(b)) antibodies labeled deposits. The anti-KP 45–54 staining was shown to colocalize with the BAM-10 anti-A*β* labeling in the plaque-like deposits (Figure 3(c)). The KP labeling was unaltered by preincubation of the antibody with NPFF [51] (Figures 3(d), 3(e), and 3(f)), and colocalization with the BAM-10 anti-A*β* labeling was still observed. The colocalization of KP and A*β* appeared to be confined to plaque-like deposits rather than throughout the tissue (Figures 3(c) and 3(f)). Control incubations with secondary antibodies showed no detectable staining or colocalization in plaque-like deposits above the background for green fluorescence (Figures 3(g) and 3(i)) or red fluorescence (Figures 3(h) and 3(i)).

### 3.4. Double-Labeling Immunohistochemistry for CRH and A*β*

**Figure 4:**
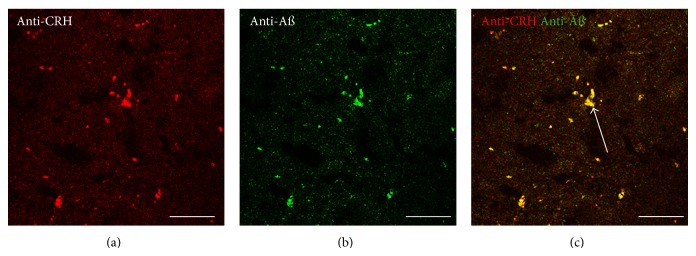
Double-labeling immunofluorescence demonstrating colocalization of CRH and A*β* in the pons in Alzheimer's disease (a–c). CRH appears red, and A*β* appears green. The overlap of CRH and A*β* appears yellow, and an example is labeled with an arrow in (c). Bars = 50 *μ*m.

To confirm the observations of Powers et al. (1987) [34] that CRH is present in AD amyloid plaques double labeling immunohistochemistry with a polyclonal anti-A*β* 21–32 antibody and the KCHMB001 monoclonal anti-CRH antibody was carried out. The results showed labeling with both antibodies and colocalization of the A*β* and CRH in plaque-like deposits (Figures 4(a), 4(b) and 4(c)). The A*β* labeling with the anti-A*β* 21–32 polyclonal primary antibody (Figure 4(b)) showed a similar pattern of A*β* labeling to that seen with the BAM-10 monoclonal anti-A*β* antibody (Figure 3(b)).

### 3.5. Double-Labeling Immunohistochemistry for Catalase and A*β*

**Figure 5:**
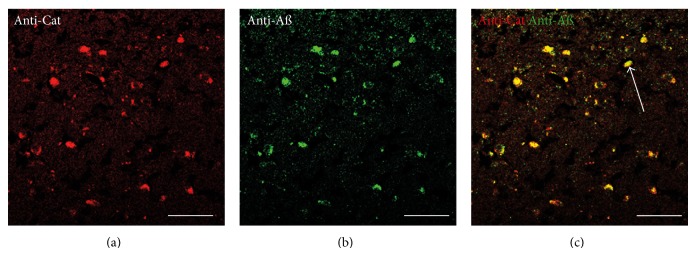
Double-labeling immunofluorescence demonstrating colocalization of catalase and A*β* in the pons in Alzheimer's disease (a–c). Catalase appears red, and A*β* appears green. The overlap of catalase and A*β* appears yellow, and an example is labeled with an arrow in (c). Bars = 50 *μ*m.

To confirm the observations of Pappolla et al. (1992) [23] that catalase is present in AD amyloid plaques double-labeling immunohistochemistry with a polyclonal anti-A*β* 21–32 antibody and the CAT-505 monoclonal anti-catalase antibody was carried out. The results showed labeling with both antibodies and colocalization of the A*β* and catalase in plaque like deposits (Figures 5(a), 5(b), and 5(c)).

### 3.6. Double-Labeling Immunohistochemistry for KP and Catalase

**Figure 6:**
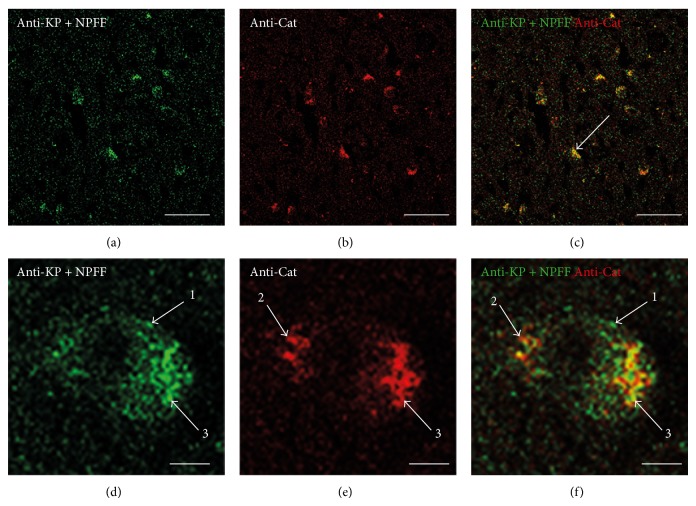
Double-labeling immunofluorescence demonstrating colocalization of KP and catalase in the pons in Alzheimer's disease (a–f). KP appears green, and catalase appears red. The overlap of KP and catalase appears yellow (merge), and an example is labeled with an arrow in (c). The arrows labeled 1 correspond to an example of KP only labeled in (d) and (f); arrows labeled 2 correspond to an example of catalase only label in (e) and (f); and arrows labeled 3 correspond to an example of KP and catalase colocalizing in (d), (e), and (f). Bars = 50 *μ*m (a)–(c) and 5 *μ*m (d)–(f).

The lack of A*β* positive deposits that did not show KP or catalase labeling suggested that the two compounds might colocalize in the plaques. Double-labeling immunohistochemistry results showed that this was the case (Figures 6(a), 6(b), and 6(c)). Of interest was the observation at higher magnification that there appeared to be specific KP and catalase sites with only limited colocalization within plaque-like deposits (Figures 6(d), 6(e), and 6(f)).

### 3.7. Fibrillar A*β* Stimulation of KP Release

**Figure 7:**
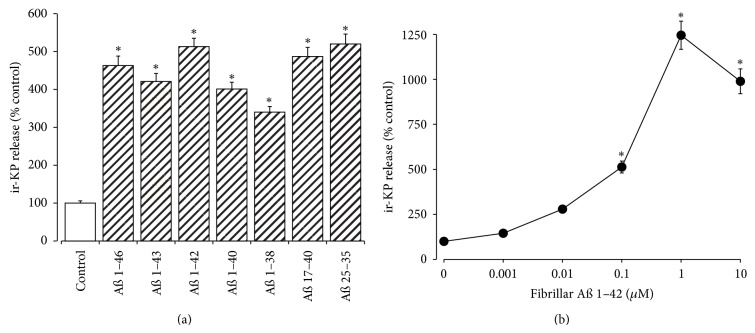
Effects of fibrillar A*β* peptides on ir-KP release from SH-SY5Y neurons. (a) Neuronal SH-SY5Y cell cultures were exposed to fibrillar A*β* 1–43, A*β* 1–42, A*β* 1–40, A*β* 1–38, A*β* 17–40, and A*β* 25–35 peptides (100 nM each) for 4 h. (b) Dose dependent release of ir-KP was determined by incubating SH-SY5Y cell cultures with fibrillar A*β* 1–42 (0–10 *μ*M) for 4 h. The release of ir-KP into the cell culture media was determined by EIA. All results, as % control (media alone), are shown as the mean ± SEM (*n* = 8) (∗: *P* < 0.05 versus control (media alone); one-way ANOVA).

The effects of fibrillar A*β* peptides on endogenous KP release were tested using human SH-SY5Y neurons. The assay used cross-reacts with KP 1–54, KP 27–54, KP 42–54, KP 45–54, and KP 45–50 but not KP 47–50 or NPFF. Results showed that fibrillar A*β* 1–46, A*β* 1–43, A*β* 1–42, A*β* 1–40, A*β* 1–38, A*β* 17–40, and A*β* 25–35 all stimulated a significant 3-4-fold increase, from a basal level of 10.5 ± 0.6 pg/mL, in ir-KP release from SH-SY5Y neurons during a 4-hour incubation (Figure 7(a)). A dose response curve for fibrillar A*β* 1–42 stimulated ir-KP release showed that at doses above 100 nM there was a significant increase in ir-KP release (Figure 7(b)). At the two highest doses (1 *μ*M and 10 *μ*M) there was significant ir-KP release; however, this was accompanied by neurotoxicity, and it is likely that the higher levels may be due to KP released from dead cells rather than KP directly stimulated by A*β*.

### 3.8. Effect of KP, CRH, and Catalase on Fibrillar A*β* 1–42 Toxicity

**Figure 8:**
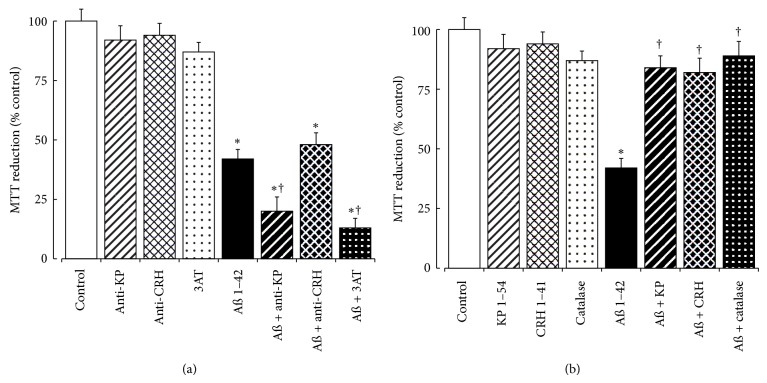
Effects of KP, CRH, and catalase on A*β* toxicity in SH-SY5Y neurons. (a) Neuronal SH-SY5Y cell cultures were exposed to 10 *μ*g/mL anti-KP 45–54 antibody (hatched columns), 10 *μ*g/mL anti-CRH antibody (cross-hatched columns), or 50 *μ*M 3AT (stippled columns) for 4 hours prior to addition of media (open columns) or fibrillar A*β* 1–42 (10 *μ*M: closed columns). (b) Neuronal SH-SY5Y cell cultures were exposed to 10 *μ*M KP 1–54 (hatched columns), 10 nM CRH (crosshatched columns), or 5 *μ*g/mL catalase (stippled columns) plus media alone (open columns) or fibrillar A*β* 1–42 (10 *μ*M: closed columns). After incubation for 16 hours, cell viability was determined by the MTT assay. All results are expressed as a % control (SH-SY5Y cells in media alone) and are expressed as the mean ± SEM (*n* = 8) (∗: *P* < 0.05 versus control (media alone); †: *P* < 0.05 versus amyloid fibrils alone; one-way ANOVA).

In order to inhibit endogenous KP, CRH, and catalase the effects of the anti-KP 45–54 antibody, the KCHMB001 anti-CRH antibody, and the catalase inhibitor 3AT on fibrillar A*β* 1–42 toxicity were tested using human SH-SY5Y neurons. The results showed that both the anti-KP 45–54 antibody and the catalase inhibitor 3AT caused a significant enhancement of A*β* 1–42 toxicity (Figure 8(a)), whilst the KCHMB001 anti-CRH antibody had no effect.

The direct effects of the KP 1–54, CRH, and catalase on fibrillar A*β* 1–42 toxicity were also tested using human SH-SY5Y neurons. The results showed that the KP 1–54, CRH, and catalase were all able to prevent A*β* neurotoxicity (Figure 8(b)).

## 4. Discussion

The colocalization of KP with A*β* in plaque-like deposits (Figure 3) is a novel observation. The colocalization of CRH with A*β* confirms the observations of Powers et al. (1987) [34] and the colocalization of catalase with A*β* confirms the observations of Pappolla et al. (1992) [23]. The failure of CRH to directly bind A*β* (Figure 1(b)) confirms previous studies [26, 31] and raises the possibility that the colocalization could be due to either the peptide being trapped within the extracellular debris, that is, part of the amyloid plaques, or that it binds to another component of the plaques. It is not possible from these studies to determine whether the KP in the A*β* positive deposits has directly bound the A*β* as described by Milton et al. (2012) [17] or whether it is trapped in the plaque debris. Both KP and catalase bind fibrillar forms of A*β* [17, 22]. The lack of secondary antibody binding to the plaque-like deposits suggests that the localization of KP, CRH catalase, and A*β* observed in these studies is due to direct binding of the primary antibodies to the respective proteins in the deposits. In the case of KP there is a known cross-reactivity of KP 45–54 antibodies with NPFF [51]; however, preadsorption of the antibody with NPFF peptide did not reduce the binding, suggesting that the KP is authentic material and not NPFF. Further studies are required to confirm these findings and also determine the regional expression of KP in AD. Changes in hypothalamic KP expression have previously been documented in women at menopause [19], and it is well known that the KiSS-1 expression is regulated by estrogen [57]. The estrogen regulation of KiSS-1 and KP peptides also differs between different brain regions [58–60], and it has been suggested that the expression patterns of the *α* and *β* estrogen receptors are responsible for this [61]. The expressions of the *α* and *β* estrogen receptors are also changed in AD [62], and this in turn may alter KiSS-1 expression and KP levels. The contribution of the cell surface G-protein-coupled estrogen receptor (GPR30) to KP regulation is unknown, but this form has been found in hippocampal tissue [63, 64] and could contribute to changes seen after menopause.

The KP peptides have been shown to be released from human SH-SY5Y neuroblastoma cells in response to fibrillar A*β* forms (Figure 7) and have previously been shown to be released by other amyloid-fibril forming peptides when in a fibrillar form [17]. The doses of amyloid-fibril forming peptides used previously were subtoxic [17] and selected based on the most toxic fibrillar amyloid peptide; in the current study we also show significant increases in KP release in response to toxic doses. The reduced release at the highest dose of A*β* tested corresponds to a significant toxicity during the incubation time and may be caused by the release of degradative enzymes from dead cells. The levels of KP released are relatively low and suggest that the neuroprotection observed with toxic doses of fibrillar A*β* is more likely to be receptor mediated rather than via a direct A*β* binding action. However, in previous studies the KP receptor antagonist or the NPFF receptor antagonist, which blocks some actions of KP [65–67], had no effect on KP neuroprotection [17]. A recent study suggests that the NPFF antagonist RF9 used in previous studies does not block all of the actions of NPFF [68] and these peptides are known to activate acid sensing ion channels (ASICs) in a nonreceptor mediated action [69]. Our observations that KP is present in the pons region of an AD brain suggest that KP neuroprotection could occur in an AD setting, and further studies into the mechanism of action of KP may define the mechanism of action.

The protective roles of catalase are well documented [12, 24–27], and here we confirm enhancement of fibrillar A*β* toxicity by the catalase inhibitor 3AT plus protection against A*β* toxicity by catalase (Figure 8). The CRH protection is also well documented [10, 28–33] and here is only seen with added CRH in the SH-SY5Y model. In the pons of AD there are both catalase and CRH colocalization with A*β* deposits, suggesting potential neuroprotective roles for catalase or CRH.

## 5. Conclusion

In conclusion, we present evidence of colocalization of KP, CRH, and catalase in A*β* positive plaque-like deposits in the pons of the AD brain. The *in vitro* observations that fibrillar A*β* stimulates ir-KP release and that both endogenous KP and catalase are neuroprotective suggest potential neuroprotective roles in AD. The colocalization study is the first demonstration of an interaction of KP with A*β* in an *in vivo* AD setting and suggests a potential role for KP in AD pathology.
